# Supplementary material for: Environment- and epigenome-wide association study of obesity in ‘Children of 1997’ birth cohort
Source: eLife. 2023 May 19;12:e82377. doi: 10.7554/eLife.82377 (PMC10198722; doi:10.7554/eLife.82377)
Supplement: Supplementary file 2. [file elife-82377-supp2.docx]

**Supplementary file 2. Associations of selected exposures with BMI after adjusting for time difference in participants of Hong Kong’s “Children of 1997” birth cohort**

| **Group Names** | **Variable description** | **With BMI at ~11.5 years** | | **With BMI at 11.5 years after time difference adjustment** | |
| --- | --- | --- | --- | --- | --- |
|  |  | **Beta** | **p value** | **Beta** | **p value** |
| Baseline characteristics | sex | 0.85 | 5.65E-22 | 0.80 | 4.37E-16 |
| Baseline characteristics | birth weight | 0.40 | 5.40E-15 | 0.40 | 1.49E-12 |
| Baseline characteristics | small for gestational age: birth weight<10% by sex and gestational week distribution in singletons | -0.68 | 9.93E-06 | -0.75 | 1.23E-05 |
| SEP | no. of smokers at home | 0.15 | 2.83E-02 | 0.30 | 1.88E-05 |
| Paternal information | paternal weight | 0.05 | 1.58E-18 | 0.05 | 8.56E-17 |
| Maternal information | maternal weight | 0.09 | 1.84E-49 | 0.10 | 7.61E-47 |
| Maternal information | postnatal smoking hygiene (i.e. second-hand smoking by timing (pre- and/or postnatal)) | 0.10 | 7.79E-05 | 0.11 | 2.97E-04 |
| Maternal information | second-hand smoke by sources and timing | 0.08 | 2.69E-05 | 0.09 | 7.69E-05 |
| Maternal information | mother exposed to second-hand smoke during pregnancy | 0.15 | 4.07E-03 | 0.16 | 7.03E-03 |
| Family history | paternal diabetes | 0.69 | 5.82E-03 | 0.77 | 5.66E-03 |
| Family history | maternal diabetes | 1.45 | 2.47E-05 | 1.38 | 1.93E-04 |
| Family history | gestational diabetes | 0.68 | 2.28E-04 | 0.62 | 2.72E-03 |
| Diet | water: frequency of consumption in the last week | 0.32 | 9.45E-06 | 0.17 | 9.60E-02 |
| Lifestyle | having meals: hours spent yesterday | -0.40 | 2.06E-03 | -0.47 | 1.01E-02 |

| **Group Names** | **Variable description** | **With BMI at 17.6 years** | | **With BMI at 17.6 years after time difference adjustment** | |
| --- | --- | --- | --- | --- | --- |
|  |  | **Beta** | **p value** | **Beta** | **p value** |
| Baseline characteristics | sex | 0.56 | 7.84E-06 | 0.48 | 7.65E-04 |
| Baseline characteristics | birth weight | 0.23 | 1.99E-03 | 0.14 | 8.28E-02 |
| Baseline characteristics | twin | -1.77 | 1.05E-03 | -1.82 | 2.75E-03 |
| Paternal information | paternal weight | 0.04 | 1.73E-09 | 0.04 | 1.95E-07 |
| Maternal information | maternal weight | 0.09 | 1.30E-28 | 0.09 | 2.81E-23 |
| Maternal information | mother exposed to second-hand smoke during pregnancy | 0.23 | 9.70E-05 | 0.12 | 1.83E-01 |
| Maternal information | postnatal smoking hygiene (i.e. second-hand smoking by timing (pre- and/or postnatal)) | 0.09 | 6.88E-02 | 0.15 | 3.33E-04 |
